# CHALLENGES AND BENEFITS OF HOSTING US STUDY ABROAD STUDENTS AT THE COLLEGE OF HEALTH AND WELFARE

**DOI:** 10.1093/geroni/igae098.2033

**Published:** 2024-12-31

**Authors:** Therése Bielsten

**Affiliations:** Jönköping University, Jönköping, Jonkopings Lan, Sweden

## Abstract

Aging in a Welfare State is a course with the aim of student exchange of knowledge about the Swedish and US healthcare systems. The campus week’s main purpose is to stimulate students’ reflection on their own country’s healthcare system. The week is initiated with a day of perspectives of and study visits on Growing up in Sweden to introduce US students to Sweden’s welfare system. However, most of the week focuses on Aging in a Western Country, Old Age Care and Perspectives of Aging. Prior to the campus week students have prepared for discussions involving a case study of the US system and a paper with reflections on current and future challenges within old age care. Discussions and comparisons of Sweden’s universal high-state regime and US mixed system for older people are a valued feature of campus week. Along with lectures, discussions and study visits related to the welfare system, leisure activities are offered in the form of excursions to Swedish sights. Planning for the campus week requires high coordination. Some of the challenges associated with coordinating campus week involve recruiting Swedish students, getting students from different countries and campuses to interact, logistics of traveling, scheduling lectures and site visits. This presentation contributes challenges and success factors for internationalization in education. Campus week is a valued feature of the course and opens opportunities for students to gain theoretical knowledge at the same time as they have the opportunity for increased understanding through real life experiences of the study visits.

